# Genetic association of anthropometric traits with type 2 diabetes in ethnically endogamous Sindhi families

**DOI:** 10.1371/journal.pone.0257390

**Published:** 2021-09-10

**Authors:** Manju Mamtani, Manisha T. Jaisinghani, Sujeet G. Jaiswal, Kanchan V. Pipal, Ashwini A. Patel, Hemant Kulkarni

**Affiliations:** 1 Lata Medical Research Foundation, Nagpur, India; 2 M&H Research, LLC, San Antonio, Texas, United States of America; University of Malaya: Universiti Malaya, MALAYSIA

## Abstract

**Background:**

Ethnically endogamous populations can shed light on the genetics of type 2 diabetes. Such studies are lacking in India. We conducted this study to determine the genetic and environmental contributions of anthropometric traits to type 2 diabetes risk in the Sindhi families in central India.

**Methods:**

We conducted a family study in Indian Sindhi families with at least one case of type 2 diabetes. Variance components methods were used to quantify the genetic association of 18 anthropometric traits with eight type 2 diabetes related traits. Univariate and bivariate polygenic models were used to determine the heritability, genetic and environmental correlation of anthropometric traits with type 2 diabetes related traits.

**Results:**

We included 1,152 individuals from 112 phenotyped families. The ascertainment-bias corrected prevalence of type 2 diabetes was 35%. Waist circumference, hip circumference and the biceps, triceps, subscapular and medial calf skinfold thicknesses were polygenically and significantly associated with type 2 diabetes. The range of heritability of the anthropometric traits and type 2 diabetes related traits was 0.27–0.73 and 0.00–0.39, respectively. Heritability of type 2 diabetes as a discrete trait was 0.35. Heritability curves demonstrated a substantial local influence of type 2 diabetes related traits. Bivariate trait analyses showed that biceps and abdominal skinfold thickness and all waist-containing indexes were strongly genetically correlated with type 2 diabetes.

**Conclusions:**

In this first study of Sindhi families, we found evidence for genetic and environmental concordance of anthropometric traits with type 2 diabetes. Future studies need to probe into the genetics of type 2 diabetes in this population.

## Introduction

While the world is rattled by the current pandemic, the more surreptitious epidemic of diabetes continues to spread [1]. Coexisting with other metabolic conditions like obesity, hypertension and dyslipidemia [2], the diabetes situation continues to worsen in developing and developed countries alike. The concomitance between type 2 diabetes and obesity [3–5] is of interest since it raises the possibility of finding simple anthropometric indexes as markers of both conditions. To that end, the association of waist circumference and type 2 diabetes is now well-established [6]. Other anthropometric indexes such as height [7], body circumferences [8] and body fat distribution [9] have been associated with type 2 diabetes to varying extent across the global populations.

Notably, stress and migration have both been shown to be associated with ‘diabesity’ [10–14]. Therefore, some ethnic groups remain at a high risk for both diabetes and obesity [15, 16]. One such ethnic group is the Sindhi population in India. According to the 2011 National Census of India (https://en.wikipedia.org/wiki/Sindhi_Hindus) there were approximately 2.8 million Sindhi individuals in India–an ethnic group with a distinctive historical background. Formation of India and Pakistan in 1947 was cursed with mass relocations across the border resulting in an exodus of Hindus living in the Sindh province (now in Pakistan) to India [17]. The need for economic independence combined with the adaptive human disposition helped these Indian Sindhi migrants settle across the length and breadth of India. Anthropometrically, the Sindhi population of India has been shown to have a unique gene pool [18, 19], facial and nasal characteristics [20, 21], and cranial measurements [22]. Whether and how the stress of migration and resettlement could have influenced the anthropometric and, subsequently, the metabolic profile of Indian Sindhi population is currently unknown.

We conducted the observational Diabetes in Sindhi Families in Nagpur (DISFIN) study (https://clinicaltrials.gov/ct2/show/NCT03918525) with the goal to estimate the prevalence of metabolic conditions in high-risk, urban Sindhi families in Nagpur–a prominently located city in central India. We aimed to test the genetic association of 18 anthropometric indexes with prevalent type 2 diabetes in the ethnically endogamous, urban Sindhi families.

## Methods

### The DISFIN study

The DISFIN is a community study of Sindhi participants enrolled from March 1, 2016 to February 28, 2017. This study was conducted in the Jaripatka, Mecosabagh and Khamla areas of Nagpur, India, where majority of the Sindhi population resides. We enrolled endogamous Sindhi families to construct family pedigrees and conduct variance components analyses on the phenotypic traits included in this study. Briefly, participants with at least one known patient of type 2 diabetes in the family were enrolled. Additional inclusion criteria were a resident of the study area; self-reported Sindhi ethnicity; age ≥20 years and who gave an informed consent. Pregnant or lactating women and patients with type 1 diabetes (known or suggested by serum C peptide assays) were excluded. All included participants provided written, informed consent. The study was approved by the Institutional Review Board of the University of Texas at Rio Grande Valley, Brownsville, Texas, USA and the Ethics Committee of the Lata Medical Research Foundation, Nagpur, India.

### DISFIN study protocol

To facilitate centralized data and sample collection, we established an Enrollment Center in each study area. These centers were established by collaboratively working with the local community. Each Enrollment Center was equipped with facilities to conduct participant interviews, clinical examination, finger prick assays as well as blood and urine sample collection. After initial registration, participants underwent an investigator-administered, detailed and structured interview about the socio-demographic characteristics. This was followed with an anthropometric investigation, thorough clinical examination and estimation of hemoglobin and random blood glucose using capillary blood. The participants then underwent a 24-hour recall dietary survey and the assessment of their physical activity using the World Health Organization’s Global Physical Activity Questionnaire (GPAQ) instrument. All interview-related and clinical examination-related data were collected using the cloud-based QuickTapSurvey application (www.quicktapsurvey.com). Thereafter, a trained phlebotomist collected blood samples for laboratory assays and storage (at -80°C) to conduct genetic investigations in future. Participants were then provided sterile urine sample collection cups. Upon receipt of these samples, dipstick assays were used to semi-quantitatively estimate urine sugar, ketones and proteins. From the remaining sample, 5ml aliquots were pipetted for cryopreservation. Reports of the laboratory investigations were distributed to the participants confidentially. Each participant was also given a diary to record his/her 7-day dietary intake with a request to return a filled-out diary after seven days.

### Anthropometric measurements

We collected data on 18 anthropometric traits that included height, weight, four circumferences (waist, hip, calf, mid-upper arm), seven skinfold thicknesses (biceps, triceps, subscapular, abdominal, suprailiac, medial calf and lateral calf), three ratios [waist-hip ratio (WHR), waist-height ratio (WHtR) and subscapular-triceps ratio (STR)] and two composite indexes [body mass index (BMI) and Durnin-Womersley body fat (D-WBF)]. Anthropometric measurements were made as per the recommendations of de Onis et al. [23]. Briefly, height was measured (to the closest mm) using a stadiometer and weight using a calibrated digital scale (to the closest 100g). All circumferences were measured using a flexible, non-stretchable tape (to the closest mm). Waist circumference (WC) was measured at the level of umbilicus; hip circumference (HC) at the level of greater trochanter; calf circumference (CC) at the largest part of the right calf; and mid-upper arm circumference (MUAC) at the midpoint between deltoid and olecranon processes. Skinfold thicknesses (SFT) were measured on the right side using a standardized (constant spring pressure of 10g/mm^2^) Harpenden caliper to the closest mm. Duplicate measurements were made at each site; in the case of a difference in measurements a third measurement was made, and the average was taken as the skinfold thickness. Data from the biceps, triceps, subscapular and suprailiac skinfold thicknesses combined with age and gender were used to derive the D-WBF percentage [24, 25].

### Laboratory investigations

In addition to the random blood glucose (RBG) measurement done at the enrolment center, we assessed the following four type 2 diabetes related traits—serum C peptide (SCP), fasting plasma glucose (FPG), fasting plasma insulin (FPI) and HbA1c. Fasting blood samples were collected in appropriate type of barcoded BD Vacutainer tubes (K-EDTA for plasma) and transported to the study laboratory using dry ice within six hours of sample collection. SCP was measured using an ultrasensitive enzyme linked immunosorbent assay [26]. FPG was determined using the hexokinase-mediated assay, FPI using an immunoenzymometric assay and HbA1c using cation-exchange high performance liquid chromatography as recommended by Reinauer et al. [27].

### Pedigree construction

Beginning with the proband, all consenting family members were interviewed about the family tree with an emphasis on enumerating as many kinships as possible. Each participant was individually interviewed for collecting the genealogical data. During the data cleaning phase, pedigrees were constructed based on the self-reported relationships. During this phase, we discovered the following errors / ambiguities: duplicate names of relatives who were not interviewed (n = 6), non-overlapping set of relationships enumerated by related individuals (n = 28), nonmatching information on alive/deceased status (n = 4), nonmatching residential address reported by different individuals (n = 8) and ambiguous endogeneity(n = 3). Next, we contacted theses shortlisted individuals again telephonically and confirmed their responses. Lastly, cases that could not be resolved using this three-step process were excluded from the family study (n = 5). The kinship2 R package (https://CRAN.R-project.org/package=kinship2) was used to construct pedigrees for visualization. This software also checked for correctness of genealogical relationships. This pedigree file was also used to create a phi-matrix in the Sequential Oligogenic Linkage Analyses Routines (SOLAR Eclipse version 8.5.1) package for further genetic analyses. However, we restricted the pedigree-based analyses to those families in which we had phenotyped at least three members.

### Sample size

The primary DISFIN study was designed to detect prevalence of type 2 diabetes at least 2% more than the then national prevalence of type 2 diabetes (6.7%) [28]. The study aimed to collect data on 1,323 individuals (assuming type I error rate of 0.05 and a power of 80%). At the conclusion of the study, adequate family-based data was available for a total of 1,152 individuals. Using the GCTA-GREML algorithm (https://shiny.cnsgenomics.com/gctaPower/) and assuming the hypothesized variance of genetic relatedness to be 0.00015 (based on our previous experience with data from San Antonio Family Heart Study [29, 30]) for a target heritability of type 2 diabetes as low as 0.3, we estimated the post hoc power of our study to be 0.8492.

### Statistical analyses

Descriptive statistics included means and standard deviations for continuous variables and proportions for categorical variables. All statistical genetic analyses were conducted in the variance components framework [31]. To ensure normal distribution, all continuous traits were inverse-normalized. Association of the anthropometric traits with type 2 diabetes was examined in a polygenic regression scenario as follows: *T* = *m* + Σ*b*_*k*_*a*_*ik*_ + *g*_*i*_ + *e*_*i*_ where, T is the discrete trait of type 2 diabetes, m is the mean, b and a are the vectors of regression coefficients and predictor trait values, g is the genetic variance component (the polygenic effect), e is the environmental variance component and i and k are the indicators for the individual and covariate indexes, respectively. All polygenic models were adjusted for potential ascertainment bias using the proband method included in the SOLAR package (http://solar-eclipse-genetics.org/index.html). Models were also adjusted for the first and second order interactions between age and sex, concomitant presence of anemia and smoking status. For this purpose, anemia was defined as hemoglobin concentration <12 g/dl for females and <13 g/dl for males [32] and smoking was defined as self-reported ever smoking.

Polygenic models for dichotomous traits used liability threshold technique. The odds ratio (OR) of a dichotomous outcome was determined as $e^{\sqrt{\pi }\beta }$ since the SOLAR software returns a negative regression coefficient from a probit model for a positively associated covariate [30]. Ascertainment bias-adjusted prevalence of a dichotomous trait was estimated from the fitted polygenic model using the standard normal transformation: φ^-1^(-m) where m is the estimated mean from the polygenic model [33]. Model fit was tested using the log likelihood statistic. The statistical significance of heritability was determined by estimating the difference between the log likelihood statistics for a sporadic (without genetic variance component) and a polygenic (with genetic variance component) model.

The classical approach of summarizing heritability as a single number may be suboptimal. Heritability can demonstrate local variation over a range of the trait values. Berentsen et al [34] have developed the concept of heritability curves in the context of twin and trio studies. We adapted this to the extended families studied here using the following method. We dichotomized a given trait at each cutoff (from trait values over a clinically meaningful range) and ran a polygenic model on the dichotomized trait at that cutoff. We then fitted a loess curve with a bandwidth of 0.4 to smoothen the heritability estimates into a heritability curve. Heritability curves were generated using dedicated Tcl/Tk scripts combined with loess smoothing.

We also conducted bivariate trait analyses to parse out the shared genetic and environmental correlation between pairs of anthropometric and type 2 diabetes-related traits as well as the anthropometric traits with each other [29, 35–37]. Overall phenotypic correlation (ρ_P_) was partitioned into genetic (ρ_G_) and environmental (ρ_E_) correlation coefficients as follows: $\rho_{P}\left(i,j\right)\;=\;\rho_{G}\left(i,j\right)\sqrt{h_{i}^{2}+h_{j}^{2}}+\rho_{E}(i,j)\sqrt{\left(1-h_{i}^{2}\right)+\left(1-h_{j}^{2}\right)}$. Here, i and j are indexes for the two traits being considered, h^2^ is the polygenic heritability, ρ_G_ is the genetic correlation coefficient, and ρ_E_ is the environmental correlation coefficient. Maximum likelihood methods were used to estimate ρ_G_ and ρ_E_. Statistical significance for a null hypothesis that ρ_G_ = 0 was tested by constraining ρ_G_ to 0 and using a chi-square test based on the difference in the log-likelihood of the unconstrained and constrained model.

Further, to determine the complex pattern of correlations among the anthropometric traits, we conducted factor analyses. In these analyses, we restricted the number of factors to those with an eigenvalue >1.0 and applied varimax rotation to the factor solution. These analyses were restricted to the measured anthropometric traits (thus excluded WHR, WHtR, STR and D-WBF which were derived traits) to avoid a Heywood case solution. All genetic association studies were conducted using the SOLAR software package, pedigree visualization was done using kinship2 package [38], descriptive statistics, factor analyses and loess curves were generated using Stata 12.0 (Stata Corp, College Station, Texas) statistical package. Statistical significance was tested at a global type I error rate of 0.05 and, where appropriate, Bonferroni’s method was used to correct for multiple testing.

## Results

### Study participants

We enrolled 1,444 participants in the DISFIN study, of whom 1,152 belonged to families with at least three phenotyped members and were included in the genetic analyses. Table 1 details the clinical, laboratory and metabolic characteristics of all the enrolled 1,444 as well as the pedigreed 1,152 participants. Briefly, the mean age was 48 years, majority of the participants were females (60%) and belonged to the upper- or lower-middle socioeconomic status (~90%). The prevalence of self-reported diabetes and hypertension was high (24% and 29%, respectively). Anemia was also highly prevalent (>45%). The characteristics of the participants included in the family study were comparable to those of the entire sample. The pedigreed participants were from 112 families–the number of phenotyped members ranging from a minimum of three to a maximum of 141. Overall, the kinship structure revealed following paired relationships: avuncular (437), parent-offspring (387), siblings (373), third degree (280), fourth degree (101), grandparent-grandchild (16), fifth degree (12) and half-siblings (5).

**Table 1:** Characteristics of the study participants.

Characteristic	All participants		Family study	
	Summary	N	Summary	N
Age [mean, (sd)] y	47.78 (13.31)	1,444	47.43 (13.41)	1,152
Females [n, (%)]	871 (60.32)	1,444	681 (59.11)	1,152
Self-reported comorbidities				
Diabetes	350 (24.24)	1,444	264 (22.92)	1,152
Hypertension	420 (29.09)	1,444	327 (28.39)	1,152
Dyslipidemia	73 (5.06)	1,444	54 (4.69)	1,152
Heart disease	89 (6.16)	1,444	68 (5.90)	1,152
Socioeconomic class (Modified Kuppuswamy scale) [n, (%)]		1,444		1,152
Upper	24 (1.66)		19 (1.65)	
Upper middle	979 (67.80)		796 (69.10)	
Lower middle	314 (21.75)		244 (21.18)	
Upper lower	124 (8.59)		92 (7.99)	
Lower	3 (0.21)		1 (0.09)	
Current smoker [n, (%)]	56 (3.88)	1,444	46 (3.99)	1,152
Anemia [n, (%)]	655 (45.52)	1,439	521 (45.42)	1,147
Laboratory Investigations				
Fasting blood glucose [mean (sd)] mg/dl	102.77 (42.76)	1,417	101.95 (42.17)	1,152
Random blood glucose [mean (sd)] mg/dl	126.68 (53.20)	1,417	125.43 (50.76)	1,152
Fasting plasma insulin [mean (sd)] IU/ml	13.88 (6.05)	1,417	13.84 (6.04)	1,152
Serum C peptide [mean (sd)] ng/ml	2.79 (4.88)	1,417	2.84 (5.39)	1,152
Hemoglobin A1c [mean (sd)] %	6.08 (1.32)	1,417	6.06 (1.29)	1,152
Metabolic conditions				
Type 2 diabetes [n, (%)]		1,410		1,152
Females	225 (26.50)		172 (25.26)	
Males	197 (35.12)		159 (33.76)	
Total	422 (29.93)		331 (28.73)	
Hypertension [n, (%)]	772 (53.46)	1,444	509 (52.86)	1,152
Dyslipidemia [n, (%)]	437 (30.84)	1,417	349 (30.30)	1,152
Obesity [n, (%)]		1,434		1,147
Indian cutoff (BMI ≥25 Kg/m^2^) [n, (%)]	1,105 (77.06)		886 (77.24)	
International cutoff (BMI ≥30 Kg/m^2^) [n, (%)]	559 (38.98)		436 (38.01)	
Central obesity [n, (%)]		1,434		1,147
Females	647 (74.71)		504 (74.23)	
Males	387 (68.13)		317 (67.74)	
Total	1,034 (72.11)		821 (71.58)	

The distribution of anthropometric measurements in the study participants is shown in Table 2. On an average, females were 14 cm shorter and nine Kg lighter as compared to males. Thus, BMI was marginally higher in females. The HC was higher in females as compared to males while MUAC, WC and CC were comparable across the genders. Consequently, WHR was lower while WHtR was higher in females. All the SFTs, except the abdominal SFT, were higher in females as compared to males. The STR was higher in males as compared to females. Strikingly, the estimated D-WBF was ~10% higher in females.

**Table 2:** Anthropometric characteristics of the study subjects.

Characteristics	All Participants		Family Study	
	Females	Males	Females	Males
	(n = 866)	(n = 568)	(n = 679)	(n = 468)
Height [mean (sd)], cm	152.04 (6.63)	166.52 (7.40)	152.51 (6.55)	166.80 (7.54)
Weight [mean (sd)], Kg	68.60 (13.44)	77.33 (14.65)	68.58 (13.38)	77.52 (14.68)
Circumferences				
Mid-upper arm [mean (sd)], cm	29.35 (4.12)	29.04 (3.87)	29.35 (4.17)	29.07 (3.87)
Waist [mean (sd], cm	93.24 (12.77)	94.85 (11.82)	92.83 (12.76)	94.62 (11.86)
Hip [mean (sd)], cm	104.56 (11.35)	100.71 (8.82)	104.30 (10.79)	100.81 (8.82)
Calf [mean (sd)], cm	33.63 (4.24)	33.94 (3.75)	33.67 (4.17)	33.92 (3.69)
Skinfold thickness				
Biceps [mean (sd)], mm	17.40 (7.58)	13.05 (6.72)	17.17 (7.67)	12.92 (6.80)
Triceps [mean (sd], mm	26.60 (6.92)	20.11 (7.41)	26.57 (6.91)	19.81 (7.20)
Subscapular [mean (sd)], mm	30.52 (9.87)	28.69 (12.42)	30.64 (9.92)	28.43 (10.02)
Abdominal [mean (sd)], mm	36.04 (11.07)	37.70 (11.81)	36.04 (11.09)	37.66 (11.72)
Suprailiac [mean (sd)], mm	31.79 (10.15)	29.81 (12.03)	31.79 (10.21)	29.51 (12.02)
Medial calf [mean (sd)], mm	30.14 (8.03)	24.97 (9.70)	24.97 (9.70)	25.07 (9.94)
Lateral calf [mean (sd)], mm	29.33 (8.88)	25.80 (9.31)	29.37 (8.86)	25.67 (9.21)
Ratios				
Waist-Hip [WHR, mean (sd)]	0.89 (0.08)	0.94 (0.07)	0.89 (0.08)	0.94 (0.07)
Waist-Height [WHtR, mean (sd)]	0.61 (0.09)	0.57 (0.07)	0.61 (0.09)	0.57 (0.07)
Subscapular-triceps [STR, mean (sd)]	1.18 (0.51)	1.53 (1.02)	1.19 (0.54)	1.51 (0.50)
Composite Indexes				
Body-mass index	29.70 (5.72)	27.90 (5.10)	29.61 (5.73)	27.88 (5.09)
[BMI, mean (sd)], Kg/m^2^				
Durnin-Womersley body fat	40.55 (4.59)	31.98 (6.47)	40.45 (4.65)	31.74 (6.45)
[D-WBF, mean (sd)], %				

### Prevalence of metabolic conditions

The overall unadjusted prevalence of type 2 diabetes was ~30% with an ~9% higher in males as compared to females (Table 1). The prevalence of general obesity as well as central obesity was >70%. Hypertension was also highly prevalent (>50%) and dyslipidemia was observed in 30% of the participants. Using the in-built proband adjustment for ascertainment bias in SOLAR, the estimated prevalence of type 2 diabetes was 35.15% [95% confidence interval (CI) 29.12%– 41.59%). Similarly, the ascertainment bias corrected prevalence of hypertension, dyslipidemia, general obesity and central obesity was 68.36% (95% CI 61.75%– 74.44%), 76.03% (95% CI 69.93%– 81.39%), 67.20% (95% CI 60.30%– 73.55%) and 29.86% (95% CI 23.94%– 36.38%), respectively. In general, therefore, the prevalence of all the metabolic conditions was high.

### Correlations among the anthropometric traits

**Fig 1:**
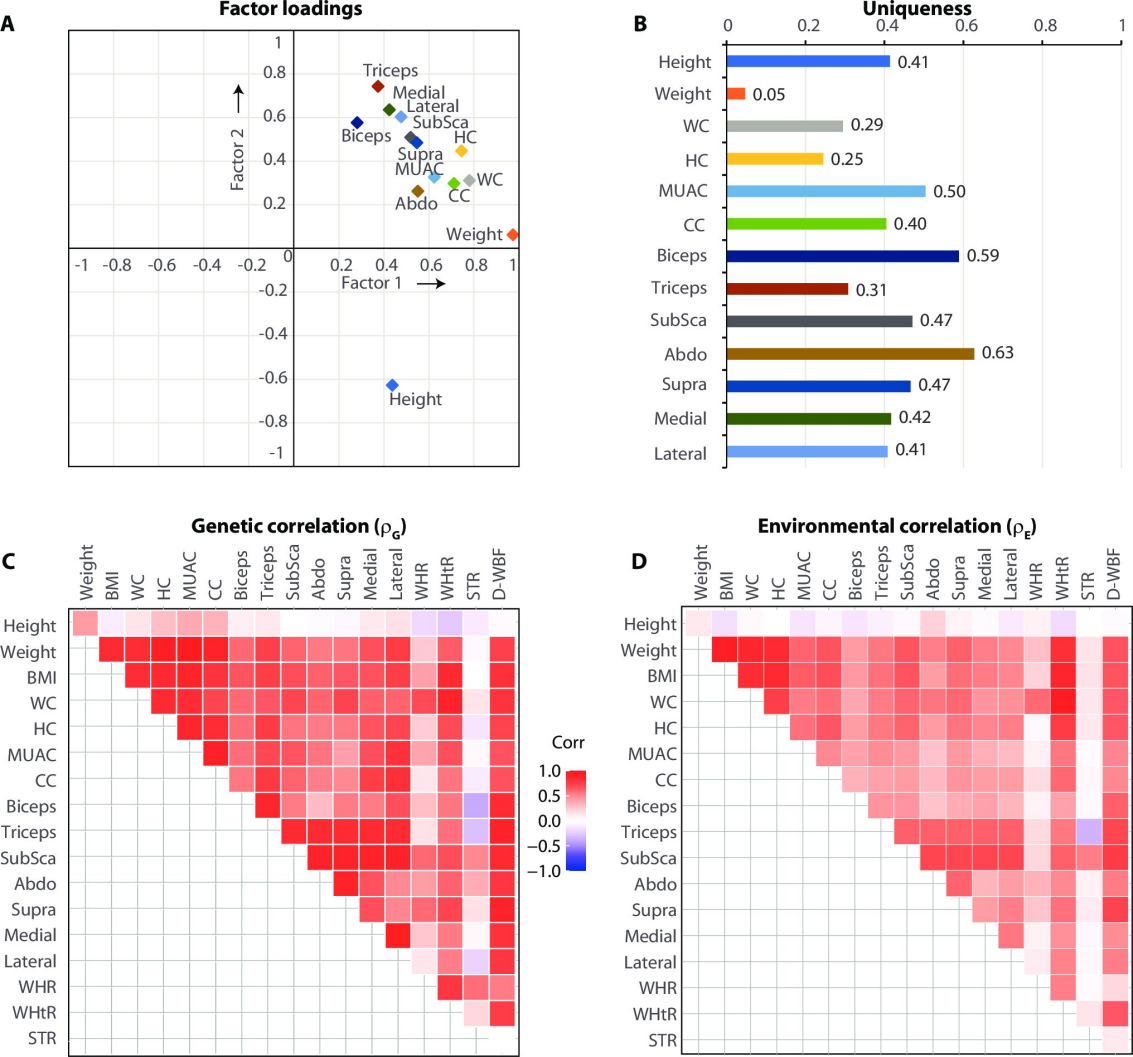
Correlations among anthropometric traits. (A-B) Results of factor analyses of the measured anthropometric traits. These analyses extracted factors with eigenvalues exceeding unity (2 factors retained) and the factor loadings (A) were estimated after varimax rotation. Panel B shows the estimated uniqueness for each anthropometric trait. In the factor analyses, derived traits such as BMI, WHR, WHtR, STR and D-WBF) were not included to avoid a Heywood case solution. (C-D) Results of bivariate trait analyses. Bivariate trait analyses quantified the genetic (C) and environmental (D) correlation coefficients which are shown in corresponding matrix plots. Color coding for plots in C and D are identical and are indicated in the color index.

Fig 1 shows the results of factor analyses using the measured anthropometric traits. The varimax rotated solution identified two significant factors that together accounted for 93.73% of overall variability in the anthropometric traits. As can be seen from Fig 1A, height, weight, all circumferences and all skinfold thicknesses represented clearly differentiable factor loading patterns. Notably, the following anthropometric traits had very high uniqueness estimates (Fig 1B): abdominal SFT (0.63), biceps SFT (0.59), MUAC (0.50) and subscapular and suprailiac SFT (0.47 each). When we explored the genetic and environmental components of the pairwise correlations using bivariate trait analyses (Fig 1C and 1D), we observed that, consistent with the results of factor analyses, the pleiotropic correlations of height and SFTs (especially biceps SFT) were low with other anthropometric traits indicating that these traits may have less genetic overlap as compared to the remaining anthropometric traits. Together, the results shown in Fig 1 show that while strong correlations existed among the anthropometric traits not all studied traits captured the same genetic or phenotypic aspects of anthropometry.

### Association of anthropometric traits with type 2 diabetes

**Fig 2:**
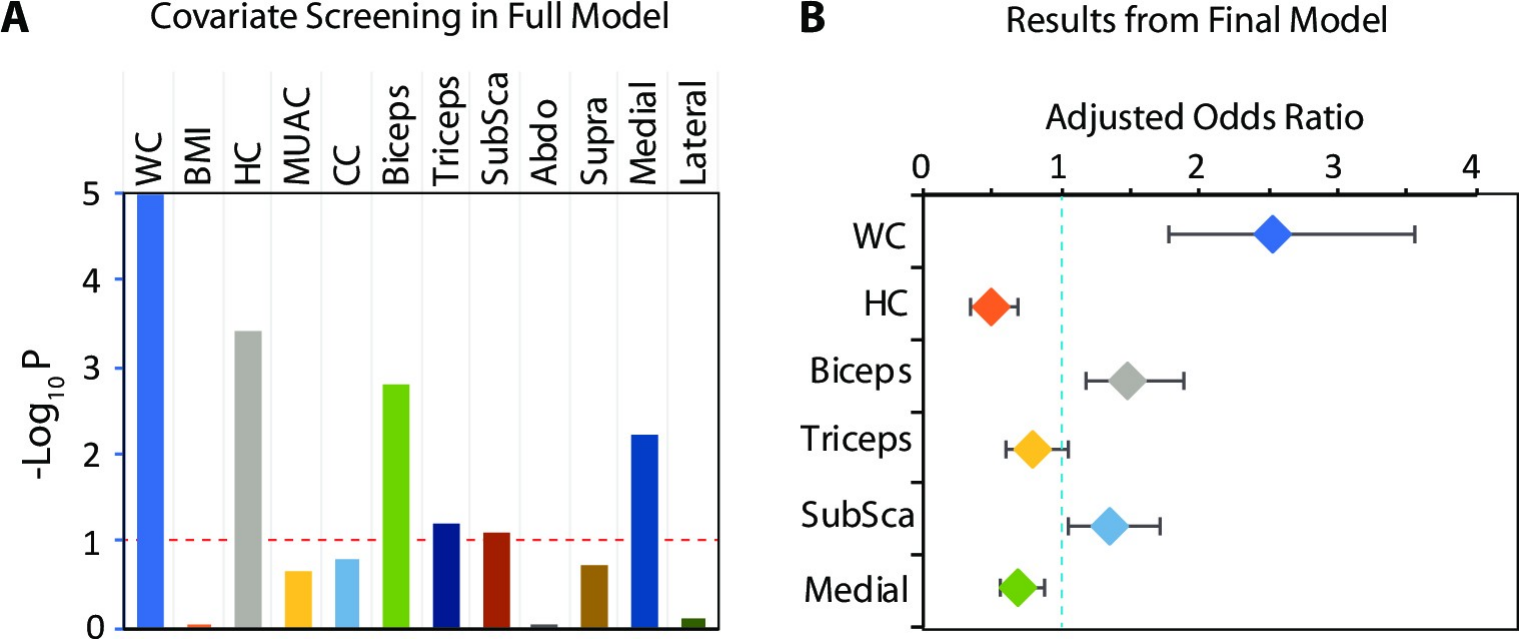
Polygenic, multivariable association of inverse-normalized anthropometric traits with type 2 diabetes. **(A)** Polygenic screening results for the full model. Ordinate represents log transformed significance values which were estimated by constraining the respective regression coefficient to 0. All variables that showed a p-value <0.1 (dashed red line) were entered into the final model. WC, waist circumference; BMI, body mass index; HC, hip circumference; MUAC, mid upper-arm circumference; CC, calf circumference. Last seven covariates in the list are skinfold thickness measurements (Subsca, subscapular; Abdo, abdominal; Supra, Suprailiac; Medial, medial calf and Lateral, lateral calf). **(B)** Results from the final model. Color-coded diamonds and error bars represent the point and 95% confidence estimates of adjusted (for first and second order interactions of age and sex, presence of anemia) odds ratio. Dashed blue line represents line of no association. Abbreviations for covariate names are the same as those in Panel A.

Using a backward elimination, stepwise polygenic regression model, we found that six anthropometric traits were retained in the final model (Fig 2A). The adjusted ORs for these six anthropometric traits were: WC– 2.52 (95% CI 1.78–3.56), p = 1.54x10^-7^; HC– 0.49 (95% CI 0.35–0.68), p = 2.56x10^-5^; biceps SFT– 1.48 (95% CI 1.17–1.88), p = 0.0013; triceps SFT– 0.79 (95% CI– 0.59–1.06), p = 0.1066; subscapular SFT– 1.34 (95% CI 1.05–1.71), p = 0.0176; and medial calf SFT– 0.69 (95% CI 0.54–0.88), p = 0.0024 (Fig 2B). Thus, a unit standard deviation increment in WC, biceps SFT and subscapular SFT was associated with an increased liability of type 2 diabetes while that of HC, medial calf SFT and triceps SFT was associated with a decreased liability of type 2 diabetes.

### Heritability of anthropometric traits

The narrow-sense heritability estimates for all anthropometric traits are shown in Table 3. All the anthropometric traits exhibited statistically significant heritability ranging from 0.20–0.72. However, after accounting for multiple test correction the statistical significance of the heritability for only MUAC was lost. The highest heritability was observed for height (0.72) while that for weight, BMI, WC and HC was in the proximity of 0.50. All the skinfold thicknesses had a heritability in the range of 0.30–0.40. Interestingly, despite higher heritability for the component anthropometric indexes, the three ratios and the D-WBF had lower estimates of heritability (0.27).

**Table 3:** Estimated narrow-sense heritability of anthropometric and type 2 diabetes related traits.

Trait/Group	h^2^r	95% CI	P_uncorr_	P_corr_
Anthropometric traits				
Height	0.73	0.60–0.86	<0.0001	<0.0001
Weight	0.52	0.36–0.68	<0.0001	<0.0001
BMI	0.48	0.31–0.65	<0.0001	<0.0001
Waist circumference	0.49	0.33–0.65	<0.0001	<0.0001
Hip circumference	0.49	0.33–0.65	<0.0001	<0.0001
Mid-upper arm circumference	0.20	0.02–0.38	0.0092	0.2706
Calf circumference	0.39	0.23–0.56	<0.0001	<0.0001
Biceps skinfold thickness	0.40	0.24–0.55	<0.0001	<0.0001
Triceps skinfold thickness	0.39	0.23–0.55	<0.0001	<0.0001
Subscapular skinfold thickness	0.29	0.14–0.45	<0.0001	0.0006
Abdominal skinfold thickness	0.28	0.14–0.42	<0.0001	0.0002
Supra iliac skinfold thickness	0.29	0.15–0.43	<0.0001	<0.0001
Medial calf skinfold thickness	0.40	0.25–0.56	<0.0001	<0.0001
Lateral calf skinfold thickness	0.38	0.22–0.54	<0.0001	<0.0001
Waist-hip ratio	0.37	0.20–0.55	<0.0001	0.0002
Waist-height ratio	0.53	0.38–0.69	<0.0001	<0.0001
Subscapular-triceps ratio	0.27	0.14–0.40	<0.0001	0.0001
Durnin-Womersley body fat	0.26	0.11–0.42	0.0002	0.0050
Type 2 diabetes related traits				
Serum C peptide				
All participants	0.06	0.00–0.21	0.1923	1.0000
Not on anti-diabetics	0.11	0.00–0.25	0.1072	1.0000
Random blood glucose				
All participants	0.24	0.09–0.38	0.0003	0.0079
Not on anti-diabetics	0.39	0.21–0.57	<0.0001	0.0005
Fasting plasma glucose				
All participants	0.21	0.05–0.36	0.0021	0.0621
Not on anti-diabetics	0.33	0.16–0.49	<0.0001	0.0026
Fasting plasma insulin				
All participants	0.10	0.00–0.24	0.0906	1.0000
Not on anti-diabetics	0.04	0.00–0.13	0.3102	1.0000
HbA1c				
All participants	0.16	0.00–0.34	0.0221	0.6658
Not on anti-diabetics	0.17	0.00–0.34	0.0448	1.0000
HOMA-IR				
All participants	0.05	0.00–0.18	0.2488	1.0000
Not on anti-diabetics	0.08	0.00–0.20	0.1707	1.0000
HOMA-β				
All participants	0.08	0.00–0.22	0.1141	1.0000
Not on anti-diabetics	0.00	0.00–0.00	0.5000	1.0000

### Heritability of type 2 diabetes related traits

As shown in Table 3, the heritability estimates for type 2 diabetes related traits were lower than those for the anthropometric traits. For example, SCP, FPI, HOMA-IR and HOMA-β all had heritability <0.10. The heritability of HbA1c was also low (0.18). In contrast, FPG and RBG had relatively higher heritability estimates which increased further (from 0.20 to 0.33 for FPG and from 0.24 to 0.39 for RBG) when the analyses were restricted to the participants who were not on anti-diabetic drugs. Such an interfering impact of anti-diabetic medication on heritability was not demonstrable for SCP, FPI, HOMA-IR, HOMA-β and HbA1c. The heritability of type 2 diabetes as a discrete trait was 0.35 (95% CI 0.03–0.66, p 0.0110) and that of self-reported type 2 diabetes was 0.31 (95% CI 0.00–0.65, p 0.0297).

### Heritability curves for quantitative traits

**Fig 3:**
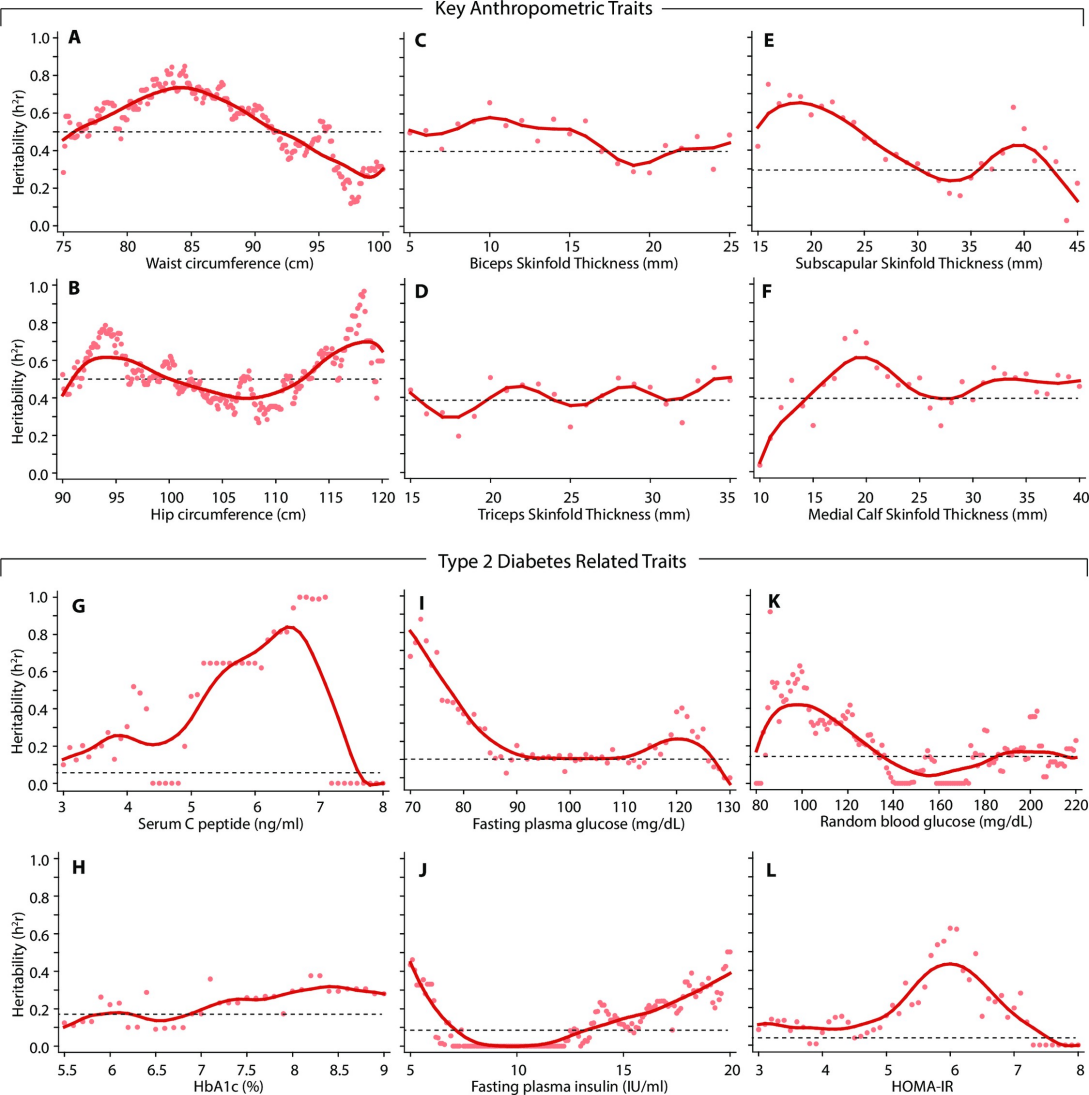
Heritability curves for significantly associated anthropometric traits (A-F) and type 2 diabetes-related traits (G-L). Heritability curves (solid red curves) represent the loess smoothed (bandwidth 0.4) curves obtained from heritability estimates estimated by dichotomizing the trait at each cutoff indicated by the pink dots. Each panel shows a dashed horizontal line that corresponds to the overall estimate of heritability (for the quantitative trait) corresponding to the values shown in Table 3.

We studied the local heritability distribution of six anthropometric traits that were significantly associated with type 2 diabetes (Fig 2B) as well as six quantitative type 2 diabetes related traits. We observed that the heritability estimates for WC and HC were clearly influenced by the respective trait values (Fig 3A and 3B) such that the heritability of WC was maximum around 85 cm while that of HC was high near the spectrum ends (<95 cm or >115 cm). Subscapular SFT also showed a heritability curve (Fig 3E) morphologically similar to that of HC. However, the heritability curves for biceps SFT, triceps SFT and medial calf SFT >20 mm showed stable estimates of heritability across trait values (Fig 3C, 3D and 3F, respectively).

The type 2 diabetes related traits showed that the heritability was highly variable over their respective ranges (Fig 3G–3L). High values of SCP, FPI and HbA1c demonstrated higher than average estimates of heritability (Fig 3G, 3J and 3H, respectively); high as well as low values of FPG and RBG had high heritability estimates (Fig 3I and 3K) while the HOMA-IR values around 6 had high heritability estimates (Fig 3L). Together, the heritability curves for the type 2 diabetes related traits showed a substantial local influence of trait values on heritability estimates.

### Bivariate trait analyses

**Fig 4:**
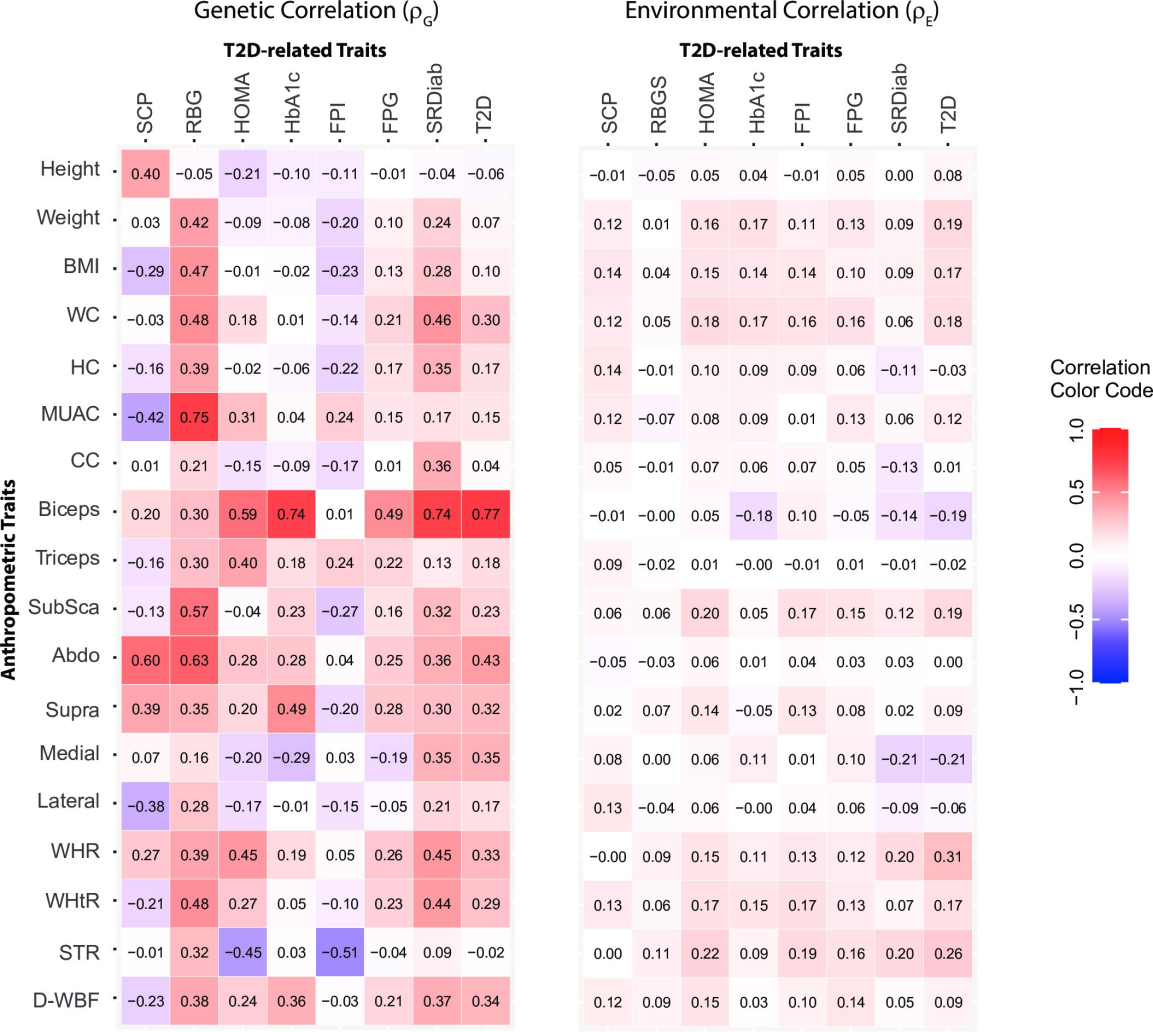
Results of bivariate trait analyses for pairs of anthropometric and type 2 diabetes related traits. Shown on the left is a color-coded heat map of the genetic correlations and on right is the heat map for corresponding environmental correlations. Color coding scheme is shown in the index on right.

Fig 4 shows the genetic and environmental correlations of the 18 anthropometric indexes with type 2 diabetes related traits as estimated using the bivariate trait analyses. Broadly, the genetic correlation coefficients were stronger in magnitude than the corresponding environmental correlation coefficients. Biceps SFT and abdominal SFT demonstrated the strongest genetic correlation with most of the type 2 diabetes related traits. For the discrete trait of type 2 diabetes, strongest genetic correlation was again observed for biceps SFT (0.77) and abdominal SFT (0.43). This was followed by the medial calf SFT, D-WBF and suprailiac SFT (~0.35). Also, the WC-containing indexes showed a genetic correlation coefficient in the vicinity of 0.30. Interestingly, general obesity- and central obesity-related anthropometric indexes (weight, BMI, WC, WHR, WHtR, subscapular SFT and STR) showed moderate to high environmental correlation. Of note, biceps SFT (-0.19) and medial calf SFT (-0.21) had strong negative environmental correlation with type 2 diabetes.

## Discussion

To our knowledge, this is the first study in ethnically endogamous urban Sindhi population of India that examined the association of anthropometric traits with type 2 diabetes in a genetic context. There are five key findings from our study: first, the prevalence of all metabolic conditions was high. In our study, there was a possibility of ascertainment bias such that we had selected families with at least one known case of type 2 diabetes. Even though we adjusted for the ascertainment bias using the proband method, the prevalence of metabolic conditions in the study population was still high. Second, the heritability of anthropometric traits was, in general, more than that of the type 2 diabetes related traits. Third, an informative pattern of local variability in heritability was demonstrated by the heritability curves of type 2 diabetes related traits. Fourth, the genetic correlation of anthropometric traits with type 2 diabetes related traits revealed a complex pattern. Lastly, biceps SFT and waist circumference were polygenically associated with type 2 diabetes; were significantly heritable; and demonstrated a strong pleiotropy with type 2 diabetes.

While we cannot directly generalize the high prevalence of metabolic conditions due to the study selection criteria, the lower limits of the 95% CIs of proband-adjusted prevalence estimates indicate that the prevalence was still high. The urban Sindhi population in India has historically faced the stress of relocation [39]. This stress combined with an energy-rich diet pattern (https://en.wikipedia.org/wiki/Sindhi_cuisine), an occupationally reduced level of physical activity [40] and the high prevalence of endocrine disruptors in the urban Indian milieu [41] have posed a theoretically increased risk of metabolic conditions. Indeed, the narrow-sense estimates of heritability of type 2 diabetes related traits was somewhat lower as compared to that reported for the Mexican American [42, 43], Caucasian American, African American, Japanese American [44, 45], Chinese [46] and middle-Eastern [47, 48] populations. Our heritability estimates were however comparable to those reported for the south Indian populations and for the Indian migrant sample residing in the United Kingdom for quantitative type 2 diabetes related traits [49, 50]. The heritability of type 2 diabetes as a discrete trait in Indian populations is unknown which we estimated to be 0.35. This observation again implies that while the genetic predisposition to type 2 diabetes in our study population is significant and undeniable, the environmental component of type 2 diabetes is also not negligible. In other populations around the world, this estimate varies in the range 0.4–0.6 [43, 44, 46, 48].

Of note, WC, biceps SFT and Subscapular SFT were associated positively while HC, triceps SFT and medial calf SFT were negatively associated with the liability of type 2 diabetes (Fig 2B). The genetic association between WC and type 2 diabetes is now well established [51, 52] and evidence is emerging for the association between biceps [53–55] and subscapular [24] SFTs with type 2 diabetes. Interestingly, HC, triceps SFT and medial calf SFT were negatively associated with the liability of type 2 diabetes. The inverse association of triceps SFT with type 2 diabetes is consistent with the positive association of subscapular-triceps ratio [56] with diabetes. Similarly, the inverse association of HC with type 2 diabetes is consistent with the positive association of waist-hip ratio with type 2 diabetes [9]. However, the association between medial calf SFT and type 2 diabetes has been less convincingly reported [54, 57]. Since the direction of the association of anthropometric measurements with type 2 diabetes varies, our results are in line with the view that body fat location [9, 58] is an informative contributor to the liability of type 2 diabetes.

The prevalence of anemia in the study population was also very high (45%). Our previous studies in this population have revealed that the prevalence of the beta-thalassemia trait (for which this is a high-risk population) ranges from 8–17% [59, 60]. It is conceivable that regular blood transfusions that can result in iron overload may partially contribute to the risk of diabetes. On the other hand, iron deficiency anemia is also known to be strongly associated with type 2 diabetes risk [61]. Further, presence of anemia can also have a direct impact on the estimation of the HbA1c [62]. Our relatively low estimates of the heritability of both type 2 diabetes as a discrete trait and HbA1c may be explained, in part, by the presence of concurrent anemia. For this reason, we statistically adjusted all the models in this study for the concomitant presence of anemia. However, some residual influence of anemia on the estimated heritability (especially of HbA1c) cannot be refuted.

Notwithstanding the potentially strong environmental contribution of lifestyle factors and comorbidities, the heritability curves demonstrated that the traditional, single-number estimates were not uniform over the range of trait values. This observation is important since it generates several interesting hypotheses for future research. First, it raises the possibility that optimal combination of type2 diabetes-related traits may be necessary to garner the genetic information. For example, high values of FPG, RBG, FPI and HbA1c may be genetically more informative than the values in the normal range. This observation resonates with the recommendation to carefully select individuals to identify functional genetic variants [63]. Second, the values of these traits in the prediabetic range had low heritability estimates implying that prediabetes may have been more environmentally than genetically dictated in the study population. Third, as the trait distribution may vary across world populations, a direct comparison of the narrow-sense heritability may be an oversimplification and underrepresentation of the genetic component of a trait. All these hypotheses will need to be specifically evaluated and tested in future studies.

Pleiotropy between anthropometric- and type 2 diabetes-related traits was especially high for the biceps SFT (*ρ*_*G*_ = 0.77) followed by abdominal SFT and waist circumference-containing indexes. All three of these indices had high uniqueness as well (Fig 1B). The genetic association of waist circumference with type 2 diabetes is now well established [6, 29, 30, 64] but the finding of the genetic link between biceps SFT and type 2 diabetes risk in this study population is novel. Of interest, we also observed a significant, negative environmental correlation between biceps SFT and type 2 diabetes. While skinfold thickness measurements have been shown to be associated with circulating glucagon levels [56], adiponectin concentrations [65], pancreatic volume [66] and the risk of incident type 2 diabetes [24]; studies on the genetic link between skinfold thickness measurements and type 2 diabetes are generally lacking. Future studies also need to consider putative ways to combine the genetic information of biceps SFT and waist circumference to better predict and understand the pathophysiology of type 2 diabetes. Together, our study results point towards the possibility of population-specific anthropometric traits that are associated with the risk and genetics of type 2 diabetes.

Our study has some limitations. First, this is a cross-sectional, observational study and the inferences drawn from this study should only be considered as genotypic and phenotypic pointers to type 2 diabetes risk rather than proof of a genetic basis. Our study was also not designed to identify any genetic and epigenetic markers of type 2 diabetes but rather provides the motivation for doing more mechanistic, genetic studies in future. Second, the prevalence estimates derived in this study should not be considered as the general population prevalence of the metabolic conditions. Our study retains its focus on the metabolic conditions and, due to the ascertainment bias, may have overestimated the disease prevalence. However, within the studied population, the prevalence of the metabolic conditions was high. Third, we did not have resources to study the cause of anemia in our population. Thus, the prevalence of beta-thalassemia trait, iron deficiency anemia and that of other possible origin could not be discerned. Regardless, since we adjusted all the models for the presence of anemia, we believe that the confounding role of anemia would be minimal in this study. Lastly, both the ethnic and kinship membership in the study were self-reported. While we took special care to identify potentially cryptic relatedness, the limitations implicit in self-reported genealogical relationships remain a limitation of the study.

Statistical genetics methods based on variance components are the first step towards a formal genetic evaluation. Family studies in ethnically endogamous populations in India are rare and this is the first such study in the Sindhi population. Our study demonstrates evidence towards both genetic and environmental basis of type 2 diabetes in the Sindhi population and shows a pleiotropic association of anthropometric traits, especially biceps SFT and waist circumference, that need to be formally investigated in future studies.
